# Graves Dermopathy With Delayed Thyrotropin Receptor Antibodies Positivity

**DOI:** 10.1210/jcemcr/luaf206

**Published:** 2025-09-25

**Authors:** Sara Ribeiro, Elisabete Rios, Julia Vide, Ana Varela

**Affiliations:** Department of Endocrinology, Diabetes and Metabolism, Centro Hospitalar Universitário São João, Porto 4200-319, Portugal; Department of Medicine, Faculty of Medicine, University of Porto, Porto 4200-319, Portugal; Department of Pathology, Unidade Local de Saúde de São João, Porto 4200-319, Portugal; Department of Pathology and Oncology, Faculty of Medicine, University of Porto, Porto 4200-319, Portugal; Department of Dermatology and Venereology, Centro Hospitalar Universitário São João, Porto 4200-319, Portugal; Department of Endocrinology, Diabetes and Metabolism, Centro Hospitalar Universitário São João, Porto 4200-319, Portugal

**Keywords:** Graves dermopathy, localized myxedema, radioiodine therapy, thyrotropin receptor antibodies

## Image Legend

**Figure 1.**
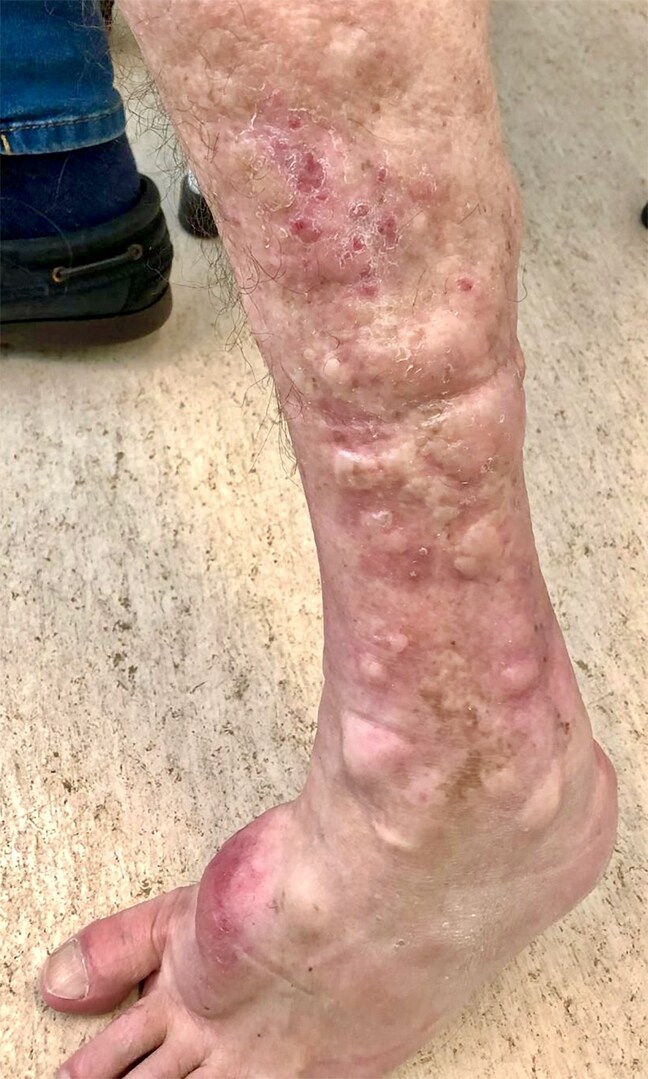
Localized myxedema with raised plaque and nodules on the pretibial area and foot dorsum.

A 52-year-old man who had smoked cigarettes since adolescence presented with thyrotoxicosis, confirmed by thyrotropin <0.001 mIU/L (<0.001 IU/L) (reference range, 0.35-4.94 mIU/L [0.35-4.94 IU/L]), free thyroxine 1.64 ng/dL (21.1 pmol/L) (reference range, 0.70-1.48 ng/dL [9.0-19.0 pmol/L]), and free triiodothyronine 10.47 pg/mL (16.1 pmol/L) (reference range, 1.71-3.71 pg/mL [2.6-5.7 pmol/L]). Thyroid scintigraphy showed diffusely increased uptake, consistent with Graves disease. Thyrotropin receptor antibodies (TRAb) were not detected at diagnosis. He had lid retraction but no active thyroid eye disease. He was treated with methimazole without adequate control and underwent radioiodine therapy 10 months later. One year after radioiodine, he developed progressively worsening, raised, painless, waxy plaques and nodules on both pretibial areas and feet (Fig. 1). Skin biopsy confirmed localized myxedema. At this point, TRAb levels were markedly elevated (>40 IU/L) (reference range, <1.8), and he remained hyperthyroid. He subsequently underwent total thyroidectomy 2 years after initial presentation. Graves dermopathy is a rare extrathyroidal manifestation, typically associated with severe ophthalmopathy and high TRAb titers [1]. This case illustrates an atypical presentation of Graves disease, with dissociation between dermopathy and orbitopathy, and delayed TRAb emergence after radioiodine, highlighting disease heterogeneity and the potential influence of smoking and immune reactivation on extrathyroidal features.
